# *Cebpd* Is Essential for Gamma-Tocotrienol Mediated Protection against Radiation-Induced Hematopoietic and Intestinal Injury

**DOI:** 10.3390/antiox7040055

**Published:** 2018-04-06

**Authors:** Sudip Banerjee, Sumit K. Shah, Stepan B. Melnyk, Rupak Pathak, Martin Hauer-Jensen, Snehalata A. Pawar

**Affiliations:** 1 Division of Radiation Health, University of Arkansas for Medical Sciences, Little Rock, AR 72205, USA; SBanerjee@uams.edu (S.B.); SSHAH3@uams.edu (S.K.S.); RPathak@uams.edu (R.P.); mhjensen@uams.edu (M.H.-J.); 2 Arkansas Children’s Hospital Research Institute, Little Rock, AR 72205, USA; MelnykStepanB@uams.edu

**Keywords:** *Cebpd*, gamma tocotrienol, granulocyte-colony stimulating factor, intestinal injury, hematopoietic injury, ionizing radiation, GSNO, GSH

## Abstract

Gamma-tocotrienol (GT3) confers protection against ionizing radiation (IR)-induced injury. However, the molecular targets that underlie the protective functions of GT3 are not yet known. We have reported that mice lacking CCAAT enhancer binding protein delta (*Cebpd^−/−^*) display increased mortality to IR due to injury to the hematopoietic and intestinal tissues and that *Cebpd* protects from IR-induced oxidative stress and cell death. The purpose of this study was to investigate whether *Cebpd* mediates the radio protective functions of GT3. We found that GT3-treated *Cebpd^−/−^* mice showed partial recovery of white blood cells compared to GT3-treated *Cebpd^+^*^/*+*^ mice at 2 weeks post-IR. GT3-treated *Cebpd^−/−^* mice showed an increased loss of intestinal crypt colonies, which correlated with increased expression of inflammatory cytokines and chemokines, increased levels of oxidized glutathione (GSSG), S-nitrosoglutathione (GSNO) and 3-nitrotyrosine (3-NT) after exposure to IR compared to GT3-treated *Cebpd*^+/+^ mice. *Cebpd* is induced by IR as well as a combination of IR and GT3 in the intestine. Studies have shown that granulocyte-colony stimulating factor (G-CSF), mediates the radioprotective functions of GT3. Interestingly, we found that IR alone as well as the combination of IR and GT3 caused robust augmentation of plasma G-CSF in both *Cebpd^+^*^/*+*^ and *Cebpd^−/−^* mice. These results identify a novel role for *Cebpd* in GT3-mediated protection against IR-induced injury, in part via modulation of IR-induced inflammation and oxidative/nitrosative stress, which is independent of G-CSF.

## 1. Introduction

The health benefits of Vitamin E are mediated through its inherent antioxidant, neuroprotective, anti-inflammatory and stress/damage response properties [1,2,3]. The Vitamin E family comprises of a set of related tocopherols and tocotrienols, collectively called tocols. The naturally occurring tocols encompass α-, β-, γ- and δ-tocopherol and α-, β-, γ- and δ-tocotrienol. Tocols and their derivatives have been extensively investigated as radiation countermeasure agents [4,5,6,7]. A number of studies have shown that tocotrienols are superior antioxidants compared to tocopherols and protect mice against radiation injury and promote post-radiation survival [8,9,10,11,12]. Several studies have shown that GT3 protects against gastrointestinal injury as well as hematopoietic injury [10,12]. The radioprotective efficacy of GT3 is mediated largely through G-CSF and helps to mobilize the progenitor cells and protects from both gastrointestinal injury as well as hematopoietic injury [13,14,15,16,17,18]. GT3 treatment is shown to suppress radiation-induced cytogenetic damage by inducing RAD50 in HUVEC cells [19]. GT3 affects the expression of a number of radiation-modulated miRNAs that are known to be involved in hematopoiesis and lymphogenesis [20]. GT3 pretreatment suppressed the upregulation of radiation-induced p53, suggesting a role in the prevention of radiation-induced damage to the spleen [20]. GT3 modulates the expression of pro-apoptotic and anti-apoptotic genes to promote intestinal stem cells and thus confer protection from radiation-induced intestinal injury [21]. However, the molecular targets of GT3 that mediate its radioprotective functions have not been elucidated.

The transcription factor CCAAT enhancer binding protein delta (*Cebpd*, C/EBPδ) is a basic leucine zipper transcription factor that is shown to regulate inflammation, oxidative stress and DNA damage response as well as innate and adaptive immune responses [22,23,24,25,26,27,28]. We have previously shown that *Cebpd*-deficiency in mice leads to IR-induced lethality to total body irradiation (TBI) [29]. Specifically, we showed that *Cebpd^−/−^* mice display impaired recovery of hematopoietic stem and progenitor cells as well as decreased white blood cells, platelets and myeloid cells, while un-irradiated mice did not show any significant phenotypic differences in these parameters [29]. We also reported that *Cebpd^−/−^* mice show decreased crypt colonies in response to 7.4 Gy, 8.5 Gy and 10 Gy TBI doses. The increased sensitivity of *Cebpd^−/−^* mouse embryonic fibroblasts (MEFs) to IR occurs due to an impaired ability to modulate IR-induced oxidative stress and mitochondrial dysfunction [28].

In this study, we investigated whether *Cebpd* mediates the radioprotective functions of GT3. Here, we report that GT3 treatment enhanced the recovery of platelets but not WBCs and neutrophils in *Cebpd^−/−^* mice. *Cebpd* is dispensable for both the IR-inducible and combination of IR and GT3-mediated induction of G-CSF. GT3 did not alleviate IR-induced oxidative/nitrosative stress and underlying intestinal injury in *Cebpd^−/−^* mice. Lastly, we also reported that the *Cebpd* gene is induced by IR, as well as by the combination of IR and GT3.

## 2. Materials and Methods

### 2.1. Animals

*Cebpd^−/−^* and *Cebpd^+/+^* mice (C57BL/6 background) were generated as described [29,30]. *Cebpd* heterozygous mice were kindly provided by Dr. Esta Sterneck (National Cancer Institute, Frederick, MD, USA) to establish a breeding colony at UAMS. 10–12-week-old male and female littermate mice, derived from heterozygous mating pairs, were used in this study. Genotyping was done as described previously [29]. The animals were housed in the Division of Laboratory Animal Medicine (University of Arkansas for Medical Sciences, Little Rock, AR, USA) under standardized conditions with controlled temperature, humidity, 12 h day and 12 h night light cycle. This study was carried out in strict accordance with the recommendations in the Guide for the Care and Use of Laboratory Animals of the National Institutes of Health. Mouse experiments were approved by the Institutional Animal Care and Use Committee. The experiments were performed under an approved protocol AUP#3611. Mice were anesthetized by isoflurane inhalation and tissues such as small intestine (proximal jejunum), femurs and tibiae and blood were harvested.

### 2.2. Irradiation and GT3 Treatment

Un-anesthetized mice were placed in mouse holders and exposed to a TBI dose of 6 Gy or 8.5 Gy in a Mark I irradiator (JL Shepherd and Associates, San Fernando, CA, USA). Dose uniformity was assessed by an independent company (Ashland Specialty Ingredients, Wilmington, DE, USA) with radiographic film and alanine tablets. Alanine tablets were analyzed by the National Institute of Standards and Technology (Gaithersburg, MD, USA) and demonstrated a dose rate of 1.09 Gy/min at 21 cm from the source. For each experiment, the dose rate was corrected for decay. GT3 was obtained from Yasoo Health Inc. (Johnson City, TN, USA). 

24 h prior to radiation exposure to 6 Gy or 8.5 Gy, *Cebpd^+^^/^^+^* and *Cebpd^−/−^* mice were injected subcutaneously either with vehicle (5% Tween-80 in normal saline), or GT3 (200 mg/kg body weight). The dose of 200 mg/kg body weight was chosen based on a previous study where it has been shown to confer 100% survival after exposure to a TBI dose of 10.5 Gy [10]. Doses higher than 200 mg/kg are shown to have a mild to moderately severe dermatitis that can be was observed clinically and microscopically in animals at the injection site [31]. Intestine (proximal jejunum) and blood samples were collected at 0, 1 h, 4 h, 24 h and 3.5 days post-irradiation. 

### 2.3. Blood Parameters

For the bone marrow injury studies, blood parameters were measured at 2 weeks post-exposure to a sublethal TBI dose of 6 Gy. Blood was collected at 0, 1, 3.5 and 7 days post-irradiation by orbital puncture, following isoflurane inhalation from untreated *Cebpd^+/+^* and *Cebpd^−/−^* mice. Blood samples were also collected from vehicle and or GT3-treated *Cebpd^+/+^* and *Cebpd^−/−^* mice at day 3.5 post-exposure to 8.5 Gy. Blood was collected in EDTA-coated tubes and blood cell parameters were determined by a Hemavet Instrument (Drew Scientific, Inc. Miami, FL, USA). Plasma samples were prepared by centrifugation at 14,000 rpm at 4 °C for 15 min and used for enzyme linked immunosorbent assay (ELISA) as described below in Section 2.7.

### 2.4. Intestinal Crypt Colony Assay

Previous studies with a LD_50_ dose of 8.5 Gy (for C57BL/6 mice) revealed that *Cebpd^−/−^* mice showed increased loss of intestinal crypt colonies at day 3.5 post-TBI and 100% mortality in the thirty-day survival study [29]. Hence in the present study, we utilized 8.5 Gy TBI to study the intestinal injury. Intestinal micro colony crypt survival assays were performed as described previously [29]. Briefly, *Cebpd^+^*^/*+*^ and *Cebpd^−/−^* mice (*n* = 7–8 per group) were sacrificed 3.5 days post-TBI dose of 8.5 Gy and segments of proximal jejunum were obtained, fixed, embedded (4–5 transverse sections per specimen), cut into 3–5-μm slices and stained with hematoxylin and eosin. A group of untreated mice of both genotypes were used as controls. The surviving crypts (those with ≥10 adjacent, chromophilic, non-Paneth cells) were counted. Four to five circumferences of proximal jejunum were scored per mouse and micro colony survival was expressed as the average number of surviving crypts per circumference. The average from each mouse was considered a single value for statistical purposes. Percent survival was calculated by normalizing to un-irradiated *Cebpd^+/+^* crypts as described previously [29].

### 2.5. Real-Time PCR

Total RNA was purified from frozen tissue using the RNeasy Plus Mini Kit (Qiagen, Valencia, CA, USA), as instructed by the manufacturer, after homogenizing the samples in TRIzol^®^ Reagent (Life Technologies, Grand Island, NY, USA). cDNA was synthesized using a cDNA reverse transcription kit (Life Technologies, Grand Island, NY, USA) after treating the RNA samples with DNase I (Qiagen, Valencia, CA, USA). Taqman assays for *Il-6* Mm00446190_m1; *Tnf-α* Mm00443258_m1; *Tgf-β* Mm01178820_m1; Mcp-1 (Ccl2), Mm00441242_m1; *Cxcl1* Mm04207460_m1; *Hmox1* Mm00516005_m1; *Nos2* Mm00440502_m1 and *Gapdh* Mm01178820_m1 were used. For *Cebpd* expression, we utilized Sybr Green-based assays. RT-PCR primers for *Cebpd* [(GAACCCGCGGCCTTCTA (F), TGTTGAAGAGGTC-GGCGA (R)] were obtained from Integrated DNA Technologies and RT² PCR Primer Set mouse *Gapdh* from Applied Biosystems (Cat # 4331182, Foster City, CA, USA). The PCR conditions followed were as described by Power Sybr Green (Cat # 1601040, Life Technologies, Austin, TX, USA) as per manufacturer’s instructions. Fold changes were calculated by normalizing to un-irradiated *Cebpd^+^^/^^+^* samples, using the standard 2^ΔΔ^*^Ct^* method as described previously [32].

### 2.6. HPLC Assays for Detection of GSH, GSSG, GSNO and 3-NT

Aliquots of intestine tissue obtained from vehicle- and or GT3-treated *Cebpd^+^^/^^+^* and *Cebpd^−/−^* mice collected at day 3.5 post 8.5 Gy were processed and analyzed by high-performance liquid chromatography (HPLC-ECD) to quantify reduced glutathione (GSH), GSSG, GSNO and 3-NT as described previously [28]. Approximately 20 mg of intestine tissue were homogenized in ice-cold phosphate-buffered saline. 10% metaphosphoric acid was added to the homogenate, incubated for 30 min on ice to precipitate the proteins. The samples were then centrifuged at 18,000× *g* at 4 °C for 15 min and 20 µL of the resulting supernatants were injected into the HPLC column for metabolite quantification, while the pellet was used for protein analysis using bicinchoninic acid protein assay. The details for HPLC elution and electrochemical detection of free unbound GSH, GSSG, GSNO and 3-NT in proteins (hydrolyzed by 6N HCl treatment) have been described previously [28,33].

### 2.7. ELISA

Plasma G-CSF was measured using the mouse G-CSF ELISA kit (Ray Biotech, GA, USA). For plasma G-CSF, the 96 well pre-coated plates were loaded with 100 μL of standard and plasma samples and processed as per the manufacturer’s instructions and absorbance was measured at 450 nm. The concentration of G-CSF in each sample was quantified against the standard curve. Values are expressed as pg/mL plasma.

### 2.8. Statistical Analyses

Statistical analyses were performed with the Graphpad prism, version 7.0 (Graphpad Software, La Jolla, CA, USA). Data are presented as mean ± S.E.M. and analyzed by one-way ANOVA followed by post-hoc analysis (*Tukey’s analysis*) or as student’s two-tailed *t* test for independent comparisons between vehicle and treated groups or between genotypes. A value of *p* < 0.05 was considered a significant difference.

## 3. Results

### 3.1. GT3-Pretreatment Showed an Impaired Recovery of WBCs, Specifically Neutrophils in Irradiated *Cebpd^−/−^* Mice

**Figure 1:**
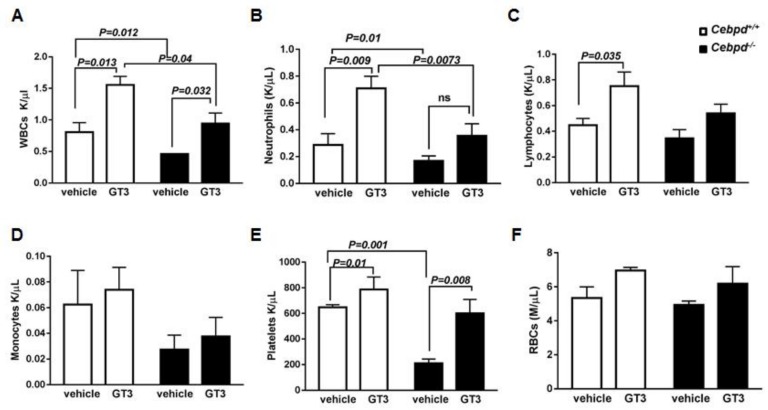
**GT3 pre-treatment showed partial rescue of the blood cells in irradiated *Cebpd******^−^^/^^−^*** **mice.** Peripheral blood cell counts (**A**) WBCs, (**B**) Neutrophils, (**C**) Lymphocytes, (**D**) Monocytes, (**E**) platelets and (**F**) RBCs from vehicle- and GT3-treated *Cebpd^+^*^/*+*^ and *Cebpd^−/−^* mice at 2 weeks after exposure to 6 Gy. Data are expressed as mean + standard error mean (S.E.M.) and obtained from *n* = 4–5 mice/genotype/group. *p <* 0.05 was considered statistically significant.

First, we investigated whether GT3 can alleviate the IR-induced hematopoietic injury in *Cebpd^−/−^* mice. Analysis of peripheral blood cells at 2 weeks post sublethal irradiation (6 Gy) revealed that the vehicle-treated *Cebpd^−/−^* mice displayed significantly reduced numbers of white blood cells (WBCs) and platelets compared to vehicle-treated *Cebpd^+^*^/*+*^ mice (Figure 1A–C), similar to our previously published study [29]. In the vehicle-treated groups, although the *Cebpd^−/−^* mice showed fewer lymphocytes and monocytes compared to *Cebpd^+^*^/*+*^ mice, these differences were not significant (Figure 1C–D).

GT3-treated *Cebpd^+^*^/*+*^ mice showed a significant increase in absolute numbers of WBCs, neutrophils, lymphocytes and platelets compared to respective vehicle- treated mice at 2 weeks post-6 Gy (Figure 1A–D). Although GT3-treatment of *Cebpd^−/−^* mice resulted in a significant increase in WBCs compared to respective vehicle-treated mice, it was significantly lower than the GT3-treated *Cebpd^+^*^/*+*^ mice. In contrast, GT3-treated *Cebpd^−/−^* mice showed a robust recovery of platelets comparable to that of GT3-treated *Cebpd^+^*^/*+*^ mice. (Figure 1E). In both vehicle- and GT3-treated groups, the numbers of RBCs were similar in both genotypes (Figure 1F). Thus, our results indicate that GT3 could rescue platelets but only provide a partial recovery of WBCs and specifically neutrophils in *Cebpd^−/−^* mice post-sublethal irradiation.

### 3.2. GT3-Treatment Protected Intestinal Crypt Colony Survival of Cebpd^+/+^ Mice but Not Cebpd^−/−^ Mice Post-TBI Exposure

Exposure of mice to TBI results in damage to the intestinal crypt stem cells [34,35]. After exposure of mice to doses in the sublethal to LD_50_ dose range of 8.5 Gy (for C57BL/6 mice), the intestinal crypt stem cells can repopulate the injured tissue. However, at doses higher than the LD_50_ dose of 8.5 Gy (9 Gy–15 Gy), there is damage to the intestinal stem cells residing in the crypts. This results in lethality primarily due to an inability to repopulate the damaged villi, which results in breakdown of the intestinal barrier leading to bacterial translocation. Intestinal stem cells are quiescent and relatively resistant to radiation; however, after radiation injury (usually 3–4 days post-irradiation), they are stimulated to enter a proliferative phase to repopulate the injured tissue and maintain tissue integrity and function and is the basis for the intestinal crypt colony survival assay [34,35]. GT3 pre-treatment has been shown to increase the intestinal crypt colony survival and protection from vascular oxidative stress and protect from acute GI syndrome [12]. 

**Figure 2:**
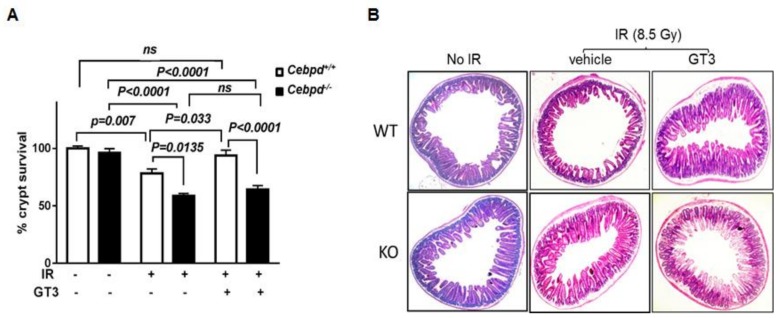
**GT3 treatment did not rescue *Cebpd******^−^^/^^−^*** **mice from radiation-induced loss of intestinal crypts.** (**A**) Surviving crypts were scored in *Cebpd^+^*^/*+*^ and *Cebpd^−/−^* mice that were un-irradiated and or vehicle- and or GT3-treated 24 h prior to exposure to 8.5 Gy. Data are expressed as mean + S.E.M. and obtained from *n* = 7–8 mice/genotype/group. *p <* 0.05 was considered statistically significant; *ns*, not significant (**B**) Representative hematoxylin and eosin stained transverse sections of small intestine (jejunum) showing the crypts and villi harvested at day 3.5 post 8.5 Gy TBI.

Here we investigated whether GT3 protects *Cebpd^+^*^/*+*^ and *Cebpd^−/−^* mice from radiation-induced intestinal injury, by examining the survival of intestinal crypt colonies at day 3.5 post-TBI. The number of surviving crypts were enumerated in GT3- and vehicle-treated *Cebdpd*^+/+^ and *Cebpd^−/−^* mice. We found that vehicle-treated *Cebpd^−/−^* mice display a significant decrease in intestinal crypt colony survival when compared to respective *Cebpd^+^*^/*+*^ mice at day 3.5 post-TBI, as shown previously [29]. *Cebpd^+^*^/*+*^ mice treated with GT3 displayed a significant increase in intestinal crypt survival compared to respective vehicle-treated mice at day 3.5 post-TBI (Figure 2A,B). However, GT3 treatment did not confer any significant improvement of intestinal crypt survival of *Cebpd^−/−^* mice when compared to respective irradiated vehicle-treated group.

### 3.3. GT3 Promotes IR-Induced Inflammatory and Oxidative Stress Markers in Cebpd^−/−^ Mice

**Figure 3:**
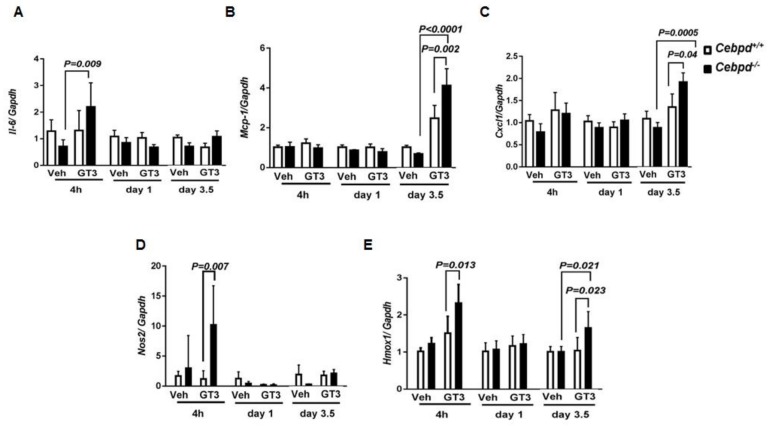
**GT3 treatment promotes IR-induced inflammation and oxidative stress in** ***Cebpd^−^^/^^−^*** **mice compared to *Cebpd^+^*^/*+*^ mice.** The intestinal expression of (**A**) *Il-6*, (**B**) *Mcp-1*, (**C**) *Cxcl1*, (**D**) *Nos2* and (**E**) *Hmox1* transcripts were analyzed at indicated time points in vehicle- and or GT3-treated *Cebpd^+^*^/*+*^ and *Cebpd^−/−^* mice exposed to 8.5 Gy. The data are expressed as fold change relative to vehicle treated *Cebpd^+^*^/*+*^ mice. The data are presented as mean + S.E.M. of *n* = 4–8 mice per treatment per genotype/time point. *p <* 0.05 was considered statistically significant.

We further examined markers of inflammatory and oxidative stress in the intestine at early and late time points post-irradiation in vehicle- and GT3-treated *Cebpd^+^*^/*+*^ and *Cebpd^−/−^* mice. GT3-treated *Cebpd^−/−^* mice showed a 2-fold increase in *Il-6* at 4 h, which decreased to basal levels comparable to the vehicle-treated controls of both genotypes at later time points (Figure 3A). While other cytokines such as *Tnf-α* and *Tgf-β* did not show any significant differences between both the genotypes in either vehicle or GT3-treated groups (Supplementary Materials Figure S1). *Tgf-β* was upregulated at day 3.5 post-TBI in vehicle- and GT3-treated mice of both genotypes (Supplementary Materials Figure S1).

Next, we examined the expression levels of chemokines, which play a prominent role in the recruitment of inflammatory cells to damaged tissues. Monocyte chemoattractant protein-1 (Mcp-1) is a chemokine that recruits monocytes and macrophages to the sites of inflammation [36]. GT3-treated *Cebpd^−/−^* mice showed a 4-fold induction of the chemokine *Mcp-1* expression compared to 2-fold induction in GT3-treated *Cebpd^+^*^/*+*^ mice at day 3.5 day post-TBI. *Cxcl1* {Chemokine (C-X-C motif) ligand 1} also known as KC is another chemokine expressed by macrophages, neutrophils and epithelial cells and has neutrophil chemoattractant activity [37]. *Cxcl1* was upregulated to 1.9-fold in *Cebpd*^−/−^ compared to 1.3-fold in *Cebpd^+^*^/*+*^ mice (Figure 3B,C).

Inducible nitric oxide synthase (*Nos2*) is a gene that is known to be induced by IR [38]. There were no significant changes in the IR-induced expression of *Nos2* in either vehicle- or GT3-treated *Cebpd^+^*^/*+*^ mice. On the other hand, GT3-treated *Cebpd^−/−^* mice showed a 10-fold induction of *Nos2* at 4 h post-irradiation, which was downregulated to basal levels by days 1 and 3.5 post-TBI (Figure 3D).

We examined the expression of heme oxygenase-1 (*Hmox1*), a well-known marker of oxidative stress [39]. GT3-treated *Cebpd^+^*^/*+*^ mice showed a 1.2-fold induction, while *Cebpd^−/−^* mice showed about a 2.3-fold induction compared to vehicle-treated *Cebpd^+^*^/*+*^ mice at 4h post-TBI (Figure 3E). The expression of *Hmox1* in the GT3 as well as vehicle-treated *Cebpd^+^*^/*+*^ mice returned to basal levels by day 1 post-TBI. In contrast compared to the vehicle-treated mice of both genotypes, we found that GT3-treated *Cebpd^−/−^* mice showed 1.6-fold upregulation compared to *Cebpd^+^*^/*+*^ mice at day 3.5 post-TBI (Figure 3E). Overall these results suggest that GT3 treatment promoted increased inflammatory and oxidative stress in *Cebpd^−/−^* mice compared to *Cebpd^+^*^/*+*^ mice in response to IR.

### 3.4. GT3-Treatment Did Not Attenuate IR-Induced Oxidative and Nitrosative Stress in Irradiated Cebpd ^−/−^ Mice

**Figure 4:**
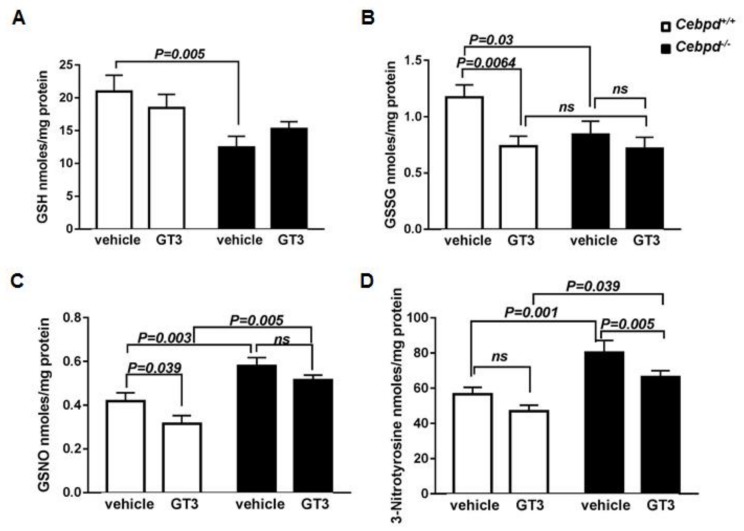
**GT3 treatment promotes increased IR-induced oxidative and nitrosative stress in *Cebpd******^−^^/^^−^*** **mice compared to *Cebpd^+^*^/*+*^ mice.** The intestinal levels of (**A**) GSH; (**B**) GSSG, (**C**) GSNO and (**D**) 3-NT were measured in intestine tissues of vehicle- or GT3- treated *Cebpd*^+/+^ and *Cebpd^−/−^* mice at day 3.5 post-exposure to 8.5 Gy (*n* = 7–8 mice/genotype/group). All the data are presented as mean + S.E.M. of *n* = 3–8 mice per treatment per genotype. *p <* 0.05 was considered statistically significant.

Next, we examined the tissue levels of the global cellular antioxidant GSH. It is known that decreased GSH is indicative of cellular oxidative stress [40,41]. The intestinal tissues from vehicle-treated *Cebpd^−/−^* mice contained 12.4 nmoles/mg protein compared to 20.96 nmoles/mg protein of GSH from respective *Cebpd^+/+^* mice at day 3.5 post-TBI (Figure 4A). The intestinal tissues from GT3-treated *Cebpd*^+/+^ mice contained showed 18.5 nmoles/mg protein, while *Cebpd^−/−^* mice showed 15.24 nmoles/mg protein of GSH. However, these differences were not significant when compared between the genotypes. Overall, GT3 treatment did not improve GSH levels in the intestine tissues in response to IR in both the genotypes.

In contrast, GT3-treatment showed a significant decrease in GSSG compared to vehicle-treatment in *Cebpd^+^*^/*+*^ mice at day 3.5 post-TBI. However, *Cebpd^−/−^* mice did not show any significant effects on GSSG levels between the vehicle- and GT3-treated groups (Figure 4B).

GSH acts as a scavenger of NO, especially when the NO levels are high enough to be detrimental to cells/tissues and forms GSNO [42]. This is particularly relevant to cells/organisms exposed to IR. GSNO is known to play a role in various inflammatory disease conditions and potentiates tissue damage [42,43,44]. Interestingly in our studies, we found a significant increase in the GSNO levels in intestines of vehicle-treated *Cebpd^−/−^* mice compared to respective *Cebpd^+^*^/*+*^ at day 3.5 post-TBI (Figure 4C). The levels of GSNO were significantly reduced in GT3-treated *Cebpd^+^*^/*+*^ mice when compared with respective vehicle-treated group at day 3.5 post-TBI. In contrast, GT3- treatment of *Cebpd^−/−^* mice did not significantly reduce the GSNO levels compared to the vehicle-treated mice (Figure 4C).

3-NT is a product of tyrosine nitration mediated by reactive nitrogen species such as peroxynitrite anion. We measured 3-NT in the intestine and found that vehicle- treated *Cebpd^−/−^* mice showed significantly increased levels compared to vehicle-treated *Cebpd^+^*^/*+*^ mice (Figure 4D). GT3-treated *Cebpd^−/−^* mice showed a significant decrease in 3-NT levels compared to respective vehicle treated group. However, GT3-treated *Cebpd^−/−^* mice showed elevated intestinal levels of 3-NT compared to respective *Cebpd^+^*^/*+*^ mice (Figure 4D). Overall these results showed that GT3 treatment led to elevated levels of GSSG, GSNO and 3-NT, which promoted the intestinal injury in irradiated *Cebpd^−/−^* mice.

### 3.5. Cebpd Is Upregulated by IR and a Combination of IR and GT3 in Intestine Tissue

**Figure 5:**
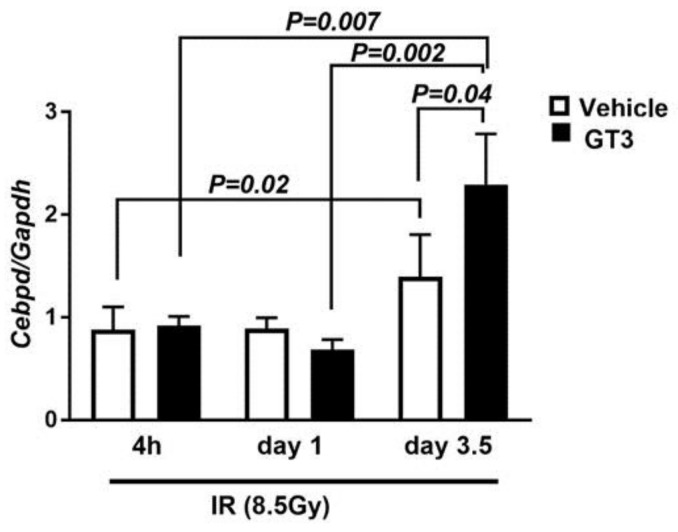
***Cebpd* is upregulated by IR and combination of IR and GT3 in the intestine tissues.** The intestinal expression of *Cebpd* and *Gapdh* transcripts were analyzed in *Cebpd^+^*^/*+*^ mice at 4 h, 1 day and 3.5 days post-exposure to 8.5 Gy. The data are expressed as fold change relative to 4 h vehicle-treated *Cebpd^+/+^* mice. The data are presented as mean + S.E.M. of *n* = 4–8 mice per treatment per genotype.

We next addressed whether IR and or GT3 stimulates the expression of *Cebpd*, which may thus play a protective role against radiation-induced intestinal injury. Here we compared the expression of *Cebpd* in intestine tissues of irradiated *Cebpd^+^*^/*+*^ mice harvested at various time points post-irradiation in vehicle- and GT3-treated mice. The expression of *Cebpd* was induced up to 1.4-fold in vehicle-treated mice and by 2.3-fold in GT3-treated mice (Figure 5). These results suggest that both IR as well as the combination of IR and GT3 stimulated the expression of *Cebpd* at day 3.5 post-TBI.

### 3.6. G-CSF Induction by IR and Combination of IR and GT3 Is Independent of Cebpd

Since GT3-mediated radiation protection has been shown to be dependent on G-CSF induction [13,14], we first examined whether impaired G-CSF induction by GT3 led to the increased IR-induced injury to hematopoietic and intestinal tissues observed in *Cebpd^−/−^* mice. Therefore, we first compared plasma G-CSF levels in *Cebpd^+^*^/*+*^ and *Cebpd^−/−^* mice at various time points post-irradiation. 

**Figure 6:**
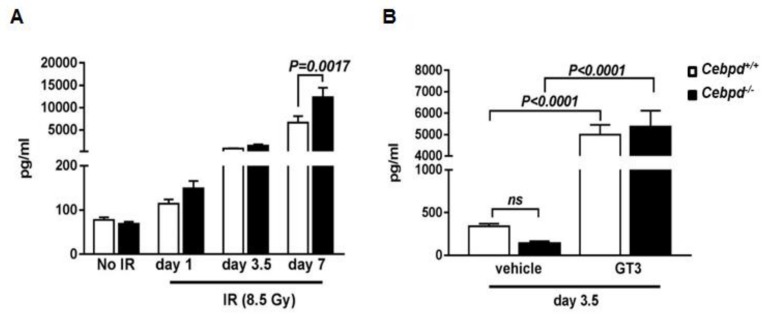
**G-CSF induction by IR and combination of IR and GT3 is independent of *Cebpd*.** (**A**) Plasma levels of G-CSF in *Cebpd^+/+^* and *Cebpd^−/−^* mice at the indicated time points post-8.5 Gy (*n* = 5/time point/genotype); and (**B**) vehicle- or GT3-treated *Cebpd^+^*^/*+*^ and *Cebpd^−/−^* mice at day 3.5 post-8.5 Gy (*n* = 7–8/genotype/group). The data are presented as mean + S.E.M. of *n* = 6–8 mice per treatment per genotype. *p <* 0.05 was considered statistically significant.

We did not find any significant differences in G-CSF levels at early time points (0–24 h) post-irradiation between both the genotypes (Supplementary Materials Figure S2). We therefore examined plasma G-CSF levels at the later time points of days 1, 3.5 and 7 post-irradiation. There were no significant differences in the plasma levels of G-CSF at day 1 post-TBI between the genotypes compared to the un-irradiated group. There was a 10.8-fold and 21.3-fold induction in the G-CSF levels in *Cebpd^+/+^* and *Cebpd^−/−^* mice compared to the respective un-irradiated mice at day 3.5 post-8.5 Gy. *Cebpd^−/−^* mice express a further 180-fold increase in plasma levels of G-CSF compared to that of an 85-fold increase in *Cebpd^+^*^/*+*^ mice at day 7 post-8.5 Gy compared to the respective un-irradiated control mice (Figure 6A). Contrary to our expectation, these results indicate that G-CSF is robustly induced in both genotypes and that *Cebpd* is not essential for the IR-induced upregulation of G-CSF.

Next, we investigated whether *Cebpd* is essential for the GT3-mediated induction of G-CSF, therefore plasma G-CSF levels in vehicle- and GT3-treated *Cebpd^+^*^/*+*^ and *Cebpd^−/−^* mice at day 3.5 post-irradiation were measured. While we found that *Cebpd^−/−^* mice express lower plasma levels of G-CSF compared to the respective vehicle-treated *Cebpd^+^*^/*+*^ mice, however this difference was not significant (Figure 6B). In the GT3-treated groups, we found a robust 14.5-fold and 39-fold induction of G-CSF in both *Cebpd^+^*^/*+*^ and *Cebpd^−/−^* mice compared to respective vehicle-treated mice (Figure 6B). These results indicate that *Cebpd* may be dispensable for the combination of IR and GT3-mediated induction of G-CSF.

## 4. Discussion

The vitamin E analog GT3 has shown promise as a radioprotector in mice and in primates [10,11]. However, the molecular targets that play a role in the radioprotective functions of GT3 remain to be elucidated. A single dose administration of GT3 24 h prior to irradiation leads to decreased radiation injury in organ systems like the bone marrow by stimulating the proliferation and differentiation of hematopoietic progenitors and intestine and vascular systems in part via reduction of vascular oxidative stress [10,12]. We found that GT3-treated *Cebpd^+^*^/*+*^ mice showed a robust rescue of WBCs, neutrophils, lymphocytes and platelets compared to vehicle-treated *Cebpd*^+/+^ mice as described previously [10]. In contrast, GT3-treated *Cebpd^−/−^* mice showed a robust rescue of platelets and WBCs but not of neutrophils compared with vehicle-treated *Cebpd^−/−^* mice. Interestingly, our previous studies had reported increased sensitivity of hematopoietic stem and progenitor cells and impaired recovery of myeloid cells in *Cebpd^−/−^* mice 2 weeks after exposure to 6 Gy, while there were no significant differences between un-irradiated *Cebpd^+/+^* and *Cebpd^−/−^* mice [29].

Depletion of intestinal stem cells (ISCs) located at or near the base of intestinal crypts post irradiation is one of the main cause of gastrointestinal syndrome [34,35,45,46]. As apical cells are shed and ISCs die or enter the cell cycle arrest due to DNA damage, the crypts become progressively denuded leading to decreased villus length, number of villi per circumference and decreased number of crypts starting about four days post-irradiation [34,35,46]. We have shown in our previous study that *Cebpd^−/−^* mice show 100% mortality after exposure to 8.5 Gy and in addition to hematopoietic injury, we also showed decreased intestinal crypt survival in the dose range of 7.4 Gy to 10 Gy [29]. GT3-treatment resulted in increased intestinal crypt colony survival of *Cebpd^+^*^/*+*^ mice compared to respective vehicle-treated mice, which is similar to a previous study [12]. However, GT3 treatment did not confer any such protective effects on crypt survival of *Cebpd^−/−^* mice. These results suggest that the protective effect of GT3 for crypt survival is dependent on *Cebpd*. Recent studies have shown that GT3 promotes intestinal cell survival via upregulation of expression of anti-apoptotic genes and downregulation of pro-apoptotic genes [21]. Our results therefore suggest that *Cebpd* may perhaps regulate GT3 mediated upregulation of anti-apoptotic genes or downregulation of pro-apoptotic genes to protect the intestinal cells in response to IR.

IR induces an inflammatory response that recruits neutrophils and macrophages to eliminate the damaged cells in the tissue and allow for tissue regeneration. Several studies have reported a role for the anti-inflammatory and antioxidant functions of GT3 [47,48,49]. We found that GT3 treatment stimulated significant increases in the expression of *Il-6* and chemokines *Cxcl1* and *Mcp-1* in *Cebpd^−/−^* mice compared to *Cebpd^+^*^/*+*^ mice, indicative of increased inflammatory stress. The main source of nitric oxide (NO) during stress conditions such as inflammation is *Nos2* and is a key player in oxidative stress [38]. We found that *the* elevated levels of *Nos2* expression in the intestines of irradiated *Cebpd^−/−^* mice, indicative of increased oxidative/nitrosative stress, were not alleviated by GT3. Another important marker of cellular oxidative stress is *Hmox1* [39,50,51]. In our studies GT3 treatment led to increased *Hmox1* expression in *Cebpd^−/−^* mice but not in *Cebpd^+^*^/*+*^ mice. *Hmox1* induction may protect cells by augmenting catabolism of pro-oxidant heme and heme proteins by the free radical scavengers under oxidative stress conditions [50]. Work by other groups has shown that GT3 downregulates both inflammation by the inhibition of NF-κB and oxidative stress by scavenging reactive oxygen species or by stabilizing Nrf2 [3,47,48,49]. Perhaps GT3-mediated *Cebpd* may modulate the IR-induced inflammation and oxidative stress, which is lacking in *Cebpd^−/−^* mice and therefore resulting in increased inflammation and oxidative stress.

GSH is known to play a key role in maintaining the redox state as well as the cellular antioxidant that imparts protection against radiation-induced oxidative stress [40,41,52]. We have earlier shown that *Cebpd* deficient mouse embryonic fibroblasts show increased basal oxidative stress due to decreased GSH levels [28]. This oxidative stress is further exacerbated by IR and is also associated with increased mitochondrial dysfunction in the cells [28]. GT3 prevented the oxidation of GSH in *Cebpd^+/+^* mice but not in the *Cebpd^−/−^* mice which showed a significant increase in the GSSG levels in the intestine tissues. These results implicate *Cebpd* as a key player in GT3-mediated GSH regeneration.

Another important player in the response of cells to IR is the reactive nitrogen species such as peroxynitrite (ONOO^−^), a product of superoxide and nitric oxide which is both reducing as well as oxidizing agent and has a very short half-life [53,54]. Several studies have implicated a role for reactive nitrogen species (RNS) in promoting radiation-induced normal tissue injury [55,56,57,58,59,60]. Various agents such as GSH, metalloporphyrins, selenium compounds, uric acid, *β*-carotene and vitamin E provide non-enzymatic protection against RNS [53]. Peroxynitrite reacts with GSH to form the nitric oxide donor GSNO [42,61]. The levels of GSNO in vehicle-treated *Cebpd^−/−^* mice were significantly higher than that in the vehicle-treated *Cebpd^+/+^* mice and were not attenuated by GT3-treatment. Overall our results indicate that GT3 was unable to protect *Cebpd^−/−^* mice from the nitrosative stress by decreasing GSNO levels. Although GT3 treatment resulted in a modest decrease in 3-NT levels but compared to GT3-treated *Cebpd^+/+^* mice, these levels were still significantly higher. Studies have shown that exogenous GSNO promotes radio sensitization [61] and this may perhaps explain the loss of intestinal crypts in *Cebpd^−/−^* mice post-irradiation.

Our studies also revealed that *Cebpd* is an IR-inducible gene that is further upregulated by combination of IR and GT3 in the intestine tissue and may perhaps explain the protective effects in *Cebpd^+/+^* mice but not in the *Cebpd^−/−^* mice. This is supported by our own studies, which reveal that *Cebpd* is induced by IR in bone marrow mononuclear cells, spleen, thymus and intestine tissues (Pawar et al., unpublished results).

Radiation is known to suppress blood neutrophil counts. G-CSF regulates the production of neutrophils within the bone marrow and stimulates neutrophil progenitor cell proliferation, differentiation and activation [62,63,64,65]. Several studies have shown that GT3 upregulates G-CSF and plays a key role in the recovery of the IR-induced bone marrow injury via mobilization of the hematopoietic progenitors in blood [13,14,17,18]. Pre-clinical studies by Kulkarni et al. (2013) demonstrated that neutralization of G-CSF abrogates GT3-mediated radiation protection, suggesting that GT3 confers radiation protection via G-CSF induction [14]. Additionally exogenous G-CSF alleviates IR-induced injury to the intestine [15,16]. In 2015, FDA approved the use of human G-CSF or filgrastim (Neupogen is the trade mark of filgrastim) as the only radiation countermeasure agent to accelerate the recovery of blood neutrophil levels after radiation exposure [64,65]. 

In the present study, we found that G-CSF was induced to similar levels in both *Cebpd^+/+^* and *Cebpd^−/−^* mice post-irradiation. Interestingly, our results reveal a G-CSF independent pathway in the radioprotection of hematopoietic and intestinal injury by GT3 that may be mediated by *Cebpd*. Similar to our findings, a recent study revealed increased bone marrow injury and lethality in *TM^P^*^ro/−^ mice, despite the expression of high G-CSF levels in response to IR and GT3 in combination [66]. There is no evidence in the literature for G-CSF as an inducer of *Cebpd*, although other family members, such as *Cebpa* and *Cebpe,* are known to be induced by G-CSF that drives myeloid differentiation [62,67]. Alternatively, it is plausible that GT3-mediated G-CSF may stimulate *Cebpd* expression cannot be ruled out. However this would need to be further confirmed by studies in G-CSF deficient mice, which is beyond the scope of the current study.

## 5. Conclusions

Overall, these studies identify *Cebpd* as a novel molecular target of GT3 that participates in its radioprotective functions via modulating IR-induced oxidative and nitrosative stress albeit in a G-CSF independent manner. The exact mechanism of *Cebpd* upregulation by GT3 and IR remains unclear. 

Future studies will delineate whether GT3 induces these changes via G-CSF or via the NF-kB and ATF3 which are modulated by GT3 [3,48,68] and are known to have binding sites in the *Cebpd* promoter region [23,69]. The expression of *Cebpd* is modulated through its 3′UTR region [70], therefore it could be plausible that GT3 mediated miRNAs could modulate the regulation of *Cebpd*. Further studies will be needed to delineate into the exact mechanism.

## Supplementary Materials

The following are available online at http://www.mdpi.com/2076-3921/7/4/55/s1, Figure S1: No significant changes in the expression of *Tnf-α* and *Tgf-β* between vehicle- and GT3-treated *Cebpd^-/-^* and *Cebpd^+/+^* mice post-irradiation. Figure S2: No significant changes in G-CSF induction by IR at early timepoint post-irradiation.
