# Supplementary figures and images for: Cebpd Is Essential for Gamma-Tocotrienol Mediated Protection against Radiation-Induced Hematopoietic and Intestinal Injury
Source: Antioxidants (Basel). 2018 Apr 6;7(4):55. doi: 10.3390/antiox7040055 (PMC5946121; doi:10.3390/antiox7040055)

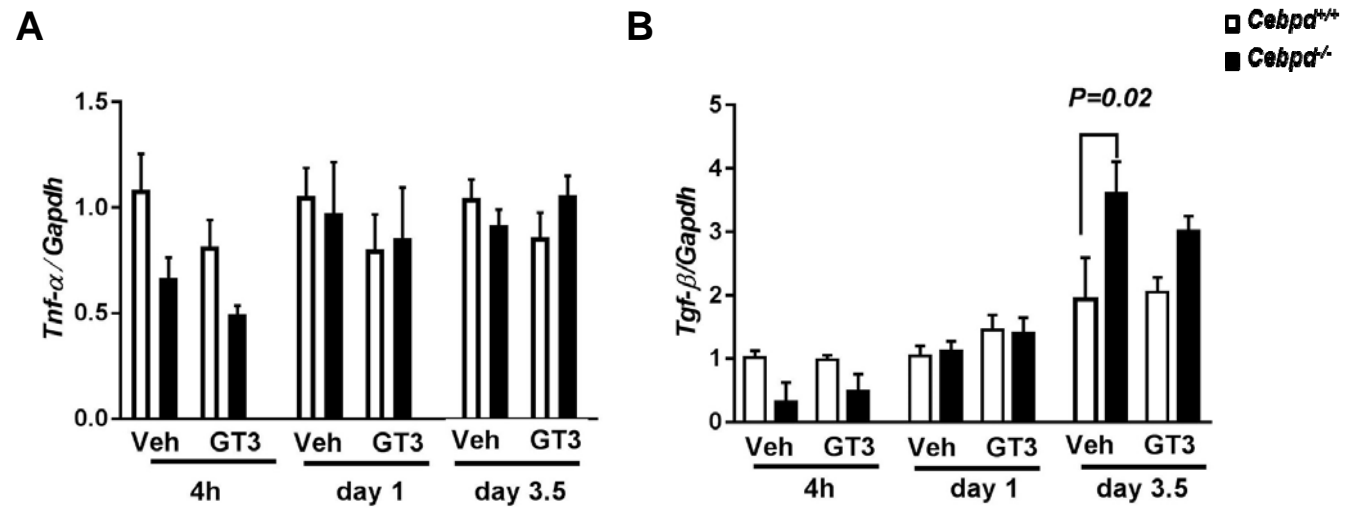

Supplementary Figure 1

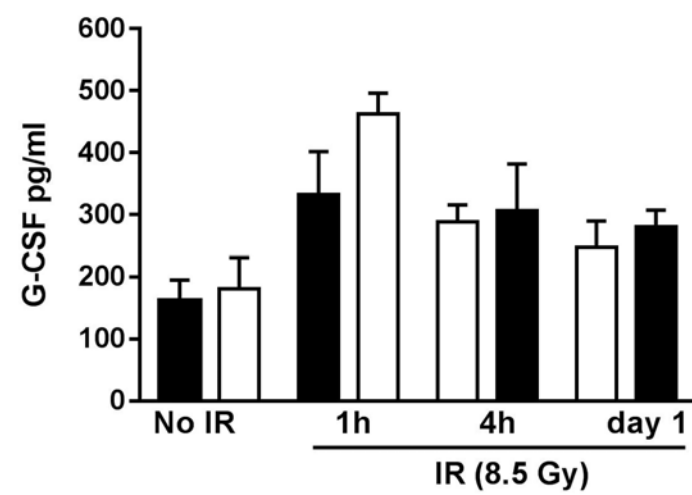

Supplementary Figure 2

Supplement: Supplementary file 1 [file antioxidants-07-00055-s001.pdf]
